# Mastering the sentence

**DOI:** 10.1007/s40037-016-0315-z

**Published:** 2016-12-08

**Authors:** Lorelei Lingard

**Affiliations:** Schulich School of Medicine & Dentistry, Health Sciences Addition, Western University, London, Ontario Canada

In the Writer’s Craft section we offer simple tips to improve your writing in one of three areas: Energy, Clarity and Persuasiveness. Each entry focuses on a key writing feature or strategy, illustrates how it commonly goes wrong, teaches the grammatical underpinnings necessary to understand it and offers suggestions to wield it effectively. We encourage readers to share comments on or suggestions for this section on Twitter, using the hashtag: #how’syourwriting?

As writers, we wordsmith tirelessly, chasing the perfect turn of phrase. We experiment with paragraph arrangement, seeking the most logical flow. But, sadly, most of us ignore our sentences. Oh, we look out for sentence fragments and other glaring errors, but we don’t think strategically about sentence structure and use it to our advantage. We should. Strong sentences are essential to good writing.

## Three types of sentence

In the English language, there are three types of sentence: simple, compound and complex. A simple sentence is made up of one independent clause, which is a clause that can stand on its own. An independent clause has a ***subject*** (a noun or noun phrase representing the main idea) and a **predicate **(a verb or verb phrase representing the main action). Simple does not mean ‘short’. Each of the following is a simple sentence:

- ***The professor*** **goes**.
- ***The professor*** **goes **reluctantly to the curriculum committee meeting.
- ***The professor*** **goes** reluctantly to the curriculum committee meeting held for the third time this semester.
- Wistfully remembering those halcyon days when decisions about teaching were hers alone, ***the professor*** **goes** reluctantly to the curriculum committee meeting held for the third time this semester.

A compound sentence is made up of two independent clauses (each with a ***subject*** and a **predicate**) that are equal in importance and can each stand alone. The following sentences illustrate how two simple sentences (1a) can be joined by a semi-colon (1b), a coordinating conjunction (1c), or a conjunctive adverb (1d) to create a compound sentence:

- 1a. ***Reviewer 1*** **liked** the paper.*** Reviewer 2*** **loathed** it.
- 1b. ***Reviewer 1*** **liked** the paper; ***Reviewer 2*** **loathed** it.
- 1c. ***Reviewer 1*** **liked** the paper, but ***Reviewer 2*** **loathed** it.
- 1d. ***Reviewer 1*** **liked** the paper; inevitably, ***Reviewer 2*** **loathed** it.

A complex sentence is made up of one independent and one dependent clause. Each clause has a **predicate**. What distinguishes complex from compound sentences is that, in complex sentences, the two clauses are not equal in importance. The ***dependent clause*** is less important and cannot stand on its own; it is signalled by a *subordinating conjunction* whose function is to join the two clauses.

***Although Reviewer 1*** **liked** ***the paper***, Reviewer 2 **loathed** it.

In this complex sentence, Reviewer 2’s loathing is given more importance than Reviewer 1’s liking due to its presence in the independent or main clause.

## The subject position

The ***subject*** is the noun or noun phrase that the **predicate** refers to, and it is the strongest ‘meaning slot’ in all three types of sentence. Therefore, that’s where your main idea should be. Imagine that you want to write about the mental health of clinical teachers.

***Many factors*** **can threaten** clinical teachers’ sanity, not least among them the hospital’s daily inpatient census.

This sentence doesn’t highlight teachers’ mental health as the main idea. Instead, ‘factors’ has the strongest prominence because it is the ***subject*** of the **predicate**. The next version places the main idea in this meaning slot to clearly communicate its importance:

***Clinical teachers’ sanity*** **can be threatened** by many factors, not least among them the hospital’s daily inpatient census.

Now that you know the power of the subject position slot, don’t waste it. Consider the next three examples in which the main idea is intended to be ‘patient complexity’. The first example distracts by making an unimportant word the ***subject*** of the **predicate**:

***Studies*** **suggest** that *patient complexity impacts teaching and learning*.

The second version weakens the main idea by using a ***nominalization*** [1]. This is a noun phrase created from other parts of speech, often a gerund (a noun created by adding ‘ing’ or ‘ed’ to a verb), and it can put the reader into cognitive overload:

***Complicated patients with multiple problems*** **impact** teaching and learning.

The third version states the main idea clearly by placing it in the most powerful meaning slot in the sentence, as ***subject*** of **the predicate**:

*Patient complexity* **impacts** teaching and learning.

## Using subject position effectively in complex sentences

Something interesting happens to the subject position as you change sentence types. A simple sentence has one predicate, and thus offers a single ***subject*** position for your main idea:

***Team communication*** is critical.

A compound sentence has two **predicates** and, therefore, two strong ***subject*** position slots.

***Team communication*** **is** critical, but ***we*** **don’t teach** it.

However, a complex sentence, while it has two **predicates**, has only one strong ***subject*** position slot – in the main clause. Therefore, if the sentence’s main idea – ‘team communication’ – gets placed in the *subordinate clause*, its importance will be diluted:

*Although team communication is critical*, ***we*** don’t teach it.

The lesson is: don’t bury your main idea in the subordinate clause of a complex sentence. It should be the ***subject*** of the *main clause*:

Although we don’t teach it, ***team communication*** *is critical*.

There are, of course, exceptions, such as when you want to deflect attention from an idea. For instance, when critically summarizing the literature, a diplomatic critique can become inadvertently personalized when the authors are in the main ***subject*** position slot:

***Carreras and Larkin*** argue that reduced duty hours do not improve resident wellness. This claim is not supported by other research in the field.

A complex sentence affords the opportunity to put Carreras and Larkin into the less prominent ***subject*** position slot in the *subordinate clause*, while still retaining the substance of the critique:

*While* ***Carreras and Larkin*** *argue that reduced duty hours do not improve resident wellness*, ***this claim*** is not supported by other research in the field.

## Topic sentences and paragraph transitions

A topic sentence is the first sentence in a paragraph, and its role is to signal the main idea that the paragraph will develop. The clearest topic sentences place that main idea in the strongest subject position slot regardless of the sentence type. Topic sentences also serve as a transitions to show why one idea/paragraph follows another. Compound and complex sentences make good transition topic sentences. In the compound sentence that follows, for example, the first clause of the sentence summarizes ***the main idea*** from the preceding paragraph, while the other clause introduces *the new idea* the current paragraph will develop:

***Competency*** has received medical educators’ attention for over a decade, but *its status as an orthodox religion* is a recent development.

## In summary

No sentence type is ‘better’ than another. Each has its place. Academic writing relies heavily on compound and complex sentences to build arguments by relating ideas. But this means that a well-placed, simple sentence can be a breath of fresh air and an easy way to highlight a key point. Master sentence structure and subject position and you will find yourself writing clearer, more coherent prose.
